# CCDC167 as a potential therapeutic target and regulator of cell cycle-related networks in breast cancer

**DOI:** 10.18632/aging.202382

**Published:** 2021-01-10

**Authors:** Pin-Shern Chen, Hui-Ping Hsu, Nam Nhut Phan, Meng-Chi Yen, Feng-Wei Chen, Yu-Wei Liu, Fang-Ping Lin, Sheng-Yao Feng, Tsung-Lin Cheng, Pei-Hsiang Yeh, Hany A. Omar, Zhengda Sun, Jia-Zhen Jiang, Yi-Shin Chan, Ming-Derg Lai, Chih-Yang Wang, Jui-Hsiang Hung

**Affiliations:** 1 Department of Biotechnology, Chia Nan University of Pharmacy and Science, Tainan 70101, Taiwan, Republic of China; 2 Department of Surgery, National Cheng Kung University Hospital, College of Medicine, National Cheng Kung University, Tainan 70101, Taiwan, Republic of China; 3 NTT Institute of Hi-Technology, Nguyen Tat Thanh University, Ho Chi Minh 700000, Vietnam; 4 Department of Emergency Medicine, Kaohsiung Medical University Hospital, Kaohsiung Medical University, Kaohsiung 80708, Taiwan, Republic of China; 5 Graduate Institute of Clinical Medicine, College of Medicine, Kaohsiung Medical University, Kaohsiung 80708, Taiwan, Republic of China; 6 Department of Biochemistry and Molecular Biology, Institute of Basic Medical Sciences, College of Medicine, National Cheng Kung University, Tainan 70101, Taiwan, Republic of China; 7 Department of Physiology, School of Medicine, College of Medicine, Kaohsiung Medical University, Kaohsiung 80708, Taiwan, Republic of China; 8 Orthopedic Research Center, College of Medicine, Kaohsiung Medical University Hospital, Kaohsiung Medical University, Kaohsiung 80708, Taiwan, Republic of China; 9 Department of Medical Research, Kaohsiung Medical University Hospital, Kaohsiung Medical University, Kaohsiung 80708, Taiwan, Republic of China; 10 Regenerative Medicine and Cell Therapy Research Center, Kaohsiung Medical University, Kaohsiung 80708, Taiwan, Republic of China; 11 Sharjah Institute for Medical Research and College of Pharmacy, University of Sharjah, Sharjah 27272, United Arab Emirates; 12 Department of Clinical Sciences, College of Pharmacy, Ajman University, Ajman 23000, United Arab Emirates; 13 Department of Pharmacology, Faculty of Pharmacy, BeniSuef University, Beni-Suef 62511, Egypt; 14 Kaiser Permanente, Northern California Regional Laboratories, The Permanente Medical Group, Berkeley, CA 94710, USA; 15 Emergency Department, Huashan Hospital North, Fudan University, Shanghai 201508, People’s Republic of China; 16 Graduate Institute of Cancer Biology and Drug Discovery, College of Medical Science and Technology, Taipei Medical University, Taipei 11031, Taiwan, Republic of China; 17 PhD Program for Cancer Molecular Biology and Drug Discovery, College of Medical Science and Technology, Taipei Medical University, Taipei 11031, Taiwan, Republic of China

**Keywords:** coiled-coil domain-containing protein 167, breast cancer, cell proliferation, cell growth, bioinformatics

## Abstract

According to cancer statistics reported in 2020, breast cancer constitutes 30% of new cancer cases diagnosed in American women. Histological markers of breast cancer are expressions of the estrogen receptor (ER), the progesterone receptor (PR), and human epidermal growth factor receptor (HER)-2. Up to 80% of breast cancers are grouped as ER-positive, which implies a crucial role for estrogen in breast cancer development. Therefore, identifying potential therapeutic targets and investigating their downstream pathways and networks are extremely important for drug development in these patients. Through high-throughput technology and bioinformatics screening, we revealed that coiled-coil domain-containing protein 167 (CCDC167) was upregulated in different types of tumors; however, the role of CCDC167 in the development of breast cancer still remains unclear. Integrating many kinds of databases including ONCOMINE, MetaCore, IPA, and Kaplan-Meier Plotter, we found that high expression levels of CCDC167 predicted poor prognoses of breast cancer patients. Knockdown of CCDC167 attenuated aggressive breast cancer growth and proliferation. We also demonstrated that treatment with fluorouracil, carboplatin, paclitaxel, and doxorubicin resulted in decreased expression of CCDC167 and suppressed growth of MCF-7 cells. Collectively, these findings suggest that CCDC167 has high potential as a therapeutic target for breast cancer.

## INTRODUCTION

Based on cancer statistics, about 30% of newly diagnosed cancers in the US are breast cancers [1]. According to Immunohistochemical staining results, breast cancer is categorized into different subtypes according to estrogen receptor (ER), progesterone receptor (PR), and human epidermal growth factor receptor (HER)-2. Up to 80% of breast cancer cases are classified as ER-positive (ER^+^), which highlights the significance of estrogen-related signaling in breast cancer [2, 3]. Currently, first-line salvage therapy for tamoxifen-resistant breast cancer patients includes fulvestrant (a selective ER down-regulator) [4, 5], cyclin-dependent kinase 4/6 (CDK4/6) inhibitors [6], aromatase inhibitors, everolimus (a mammalian target of rapamycin inhibitor) [7], and histone deacetylase (HDAC) inhibitors [8]. However, resistance to these salvage therapies ultimately develops, and patients die from their cancer [9, 10]. Therefore, it is important to explore new effective therapeutics for ER^+^ breast cancer patients. Coiled-coil domain (CCD) constituents are alpha-helix motifs expressed in different types of proteins. Owing to structural flexibility, they can function in various biological processes, including cell proliferation, migration, and signal transduction [11, 12]. In recent publications, CCD-containing (CCDC) proteins were aberrantly activated in multiple types of tumors, including CCDC178 in liver cancer, CCDC88A in pancreatic cancer, and CCDC8 in lung cancer [13–18]. In addition, CCDC106 promotes the proliferation of lung cancer cell lines, and CCDC34 contributes to colorectal cancer development by inhibiting apoptosis signaling and promoting invasion [19]. Inhibition of CCDC69 increases platinum-induced apoptosis in A2780 ovarian cancer cells [20]. Knockdown of CCDC106 enhances apoptosis and suppresses growth of MCF7 breast cancer by stabilizing p53 [21]. However, there is yet little knowledge of the role of CCDC167 in breast cancer development.

Differential gene expression analyses in high-throughput techniques, such as RNA-sequencing (RNA-Seq), are performed by comparing gene expression profiles between two different conditions. The results define a set of genes with high expressions in cancer and low expressions in normal tissue through statistical tests [22–26]. However, in some particular circumstances, non-differentially expressed genes can contribute to disease dysfunction through clusters of co-expressed genes. These genes may manifest their functions through interaction networks with other differentially expressed genes. Therefore, expression signatures of these co-expressed genes might be crucial factors indirectly affecting the disease condition [27–31]. In the present study, we used a meta-analysis approach combined with a literature review to explore potential therapeutic targets in breast cancer. We systematically investigated messenger (m)RNA expression of CCDC167 and the survival probability of ER^+^ breast cancer patients using a public high-throughput database. Meanwhile, we also investigated the effects of CCDC167 on the progression of breast cancer with an experimental approach.

## RESULTS

### Increased expression of CCDC167 in breast cancer patients

**Figure 1:**
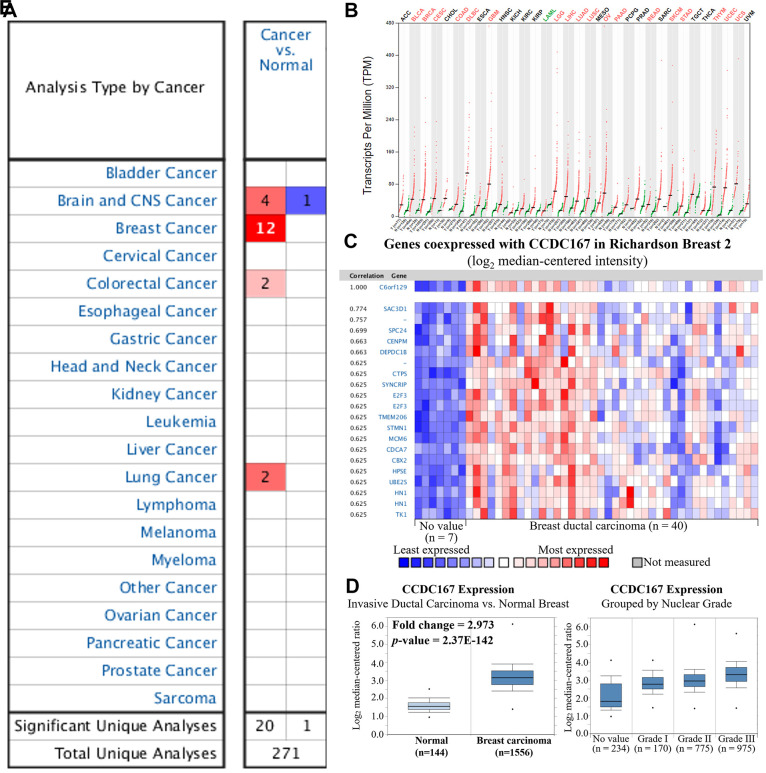
**Gene expression profiles of coiled-coil domain-containing protein 167 (CCDC167) in normal breast tissues and breast cancer.** (**A**) Expression levels of CCDC167 in different types of cancers compared to normal tissues. The CCDC167 gene was found to have upregulated expression in various tissue types, and the color gradient represents a lower gene rank percentile. (**B**) CCDC167 expression level from 33 cancer datasets with transcripts per million (TPM) levels using the GEPIA database. Significant CCDC167 overexpression in cancers is highlighted in red, including the BRCA dataset, whereas downregulation is labeled in green. (**C**) Co-expression patterns of CCDC167 in the Oncomine database. CCDC167 is also called C6orf129. (**D**) CCDC167 expression was higher relative to normal breast tissues in the METABRIC database. (**E**) Correlations between CCDC167 and histological differentiation of breast cancer, with increasing expression levels of CCDC167 as tumors progressed from low to high grade.

Transcription expression levels of CCDC167 in 20 types of cancer were screened in the Oncomine database. The CCDC167 gene was upregulated in breast cancer compared to normal tissues in 12 studies (Figure 1A). Furthermore, the GEPIA database was used to compare the CCDC167 gene in normal and cancerous tissues from 33 TCGA RNA-Seq datasets. We found that CCDC167 expression was upregulated in 18 of 33 datasets, including an invasive breast carcinoma (BRCA) dataset (Figure 1B). In the Richardson dataset, the CCDC167 gene and its co-expressed genes, such as *SAC3D1*, *SPC24*, *CENPM* and *DEPDC1B*, were substantially upregulated in breast ductal carcinoma compared to the normal group (Figure 1C). A similar expression profile of CCDC167 was also found in the METABRIC dataset for invasive ductal carcinoma versus normal breast tissues (Figure 1D) and other subtypes (Supplementary Figure 1). Moreover, there was a correlation between CCDC167 and histological differentiation of breast cancer, with increasing expression levels of CCDC167 as tumors progressed from nuclear grade I to III (Figure 1E). The bioinformatics analysis of breast cancer patient samples collectively provided clues to a potential role of CCDC167 during breast cancer development.

### CCDC167 regulates cancer development via cell cycle-related pathways

**Figure 2:**
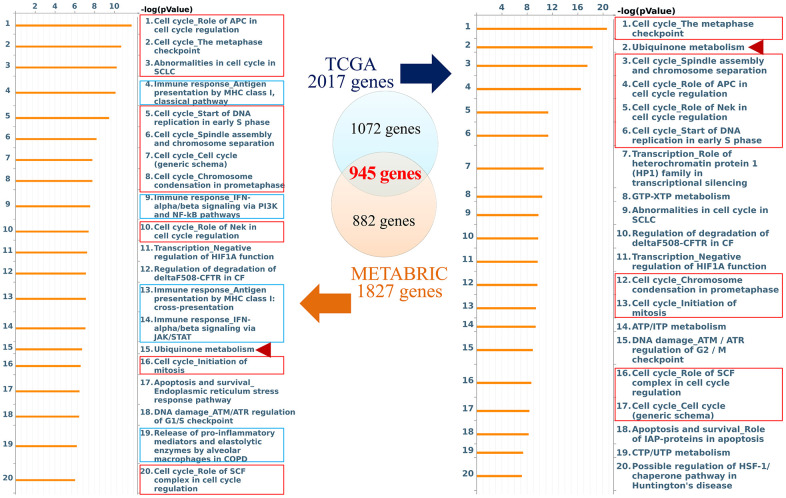
**Networks from coiled-coil domain-containing protein 167 (CCDC167)-co-expressed genes in breast cancer.** The Venn diagram circles represent co-expressed genes of CCDC167 from each database; the blue circle represents co-expressed genes in TCGA database, and the orange circle represents co-expressed genes in the METABRIC database. The MetaCore signaling pathway analysis demonstrated that the “cell cycle role of anaphase-promoting complex (APC) in cell cycle regulation” and cell cycle-related signaling (red rectangles) were significantly correlated with CCDC167 gene expression. Immune-response signaling is marked with blue rectangles, and ubiquinone metabolism is scored with red arrowheads.

The role of CCDC167 in breast cancer has been less well studied, therefore, in the present study, we used co-expression analyses to reveal biological functions and information of possible mechanisms. Co-expression profiles of CCDC167 from breast cancers were identified in the METABRIC and TCGA databases. MetaCore analysis of GO enrichment was performed to predict gene functions and regulatory patterns (Figure 2). We merged results from METABRIC and TCGA to obtain common co-expressed genes, which were then imported into the MetaCore platform. Results of pathway maps indicated that cell cycle-related signaling plays an essential role in breast cancer according to both databases (Supplementary Table 1). We discovered that genes co-expressed with CCDC167 also affected immune- and ubiquinone-related pathways, including “immune response antigen presentation by MHC class I”, “immune response interferon (IFN)-alpha/beta signaling via phosphatidylinositol 3-kinase (PI3K) and nuclear factor (NF)-κB pathways”, and “ubiquinone metabolism” in breast cancer.

**Figure 3:**
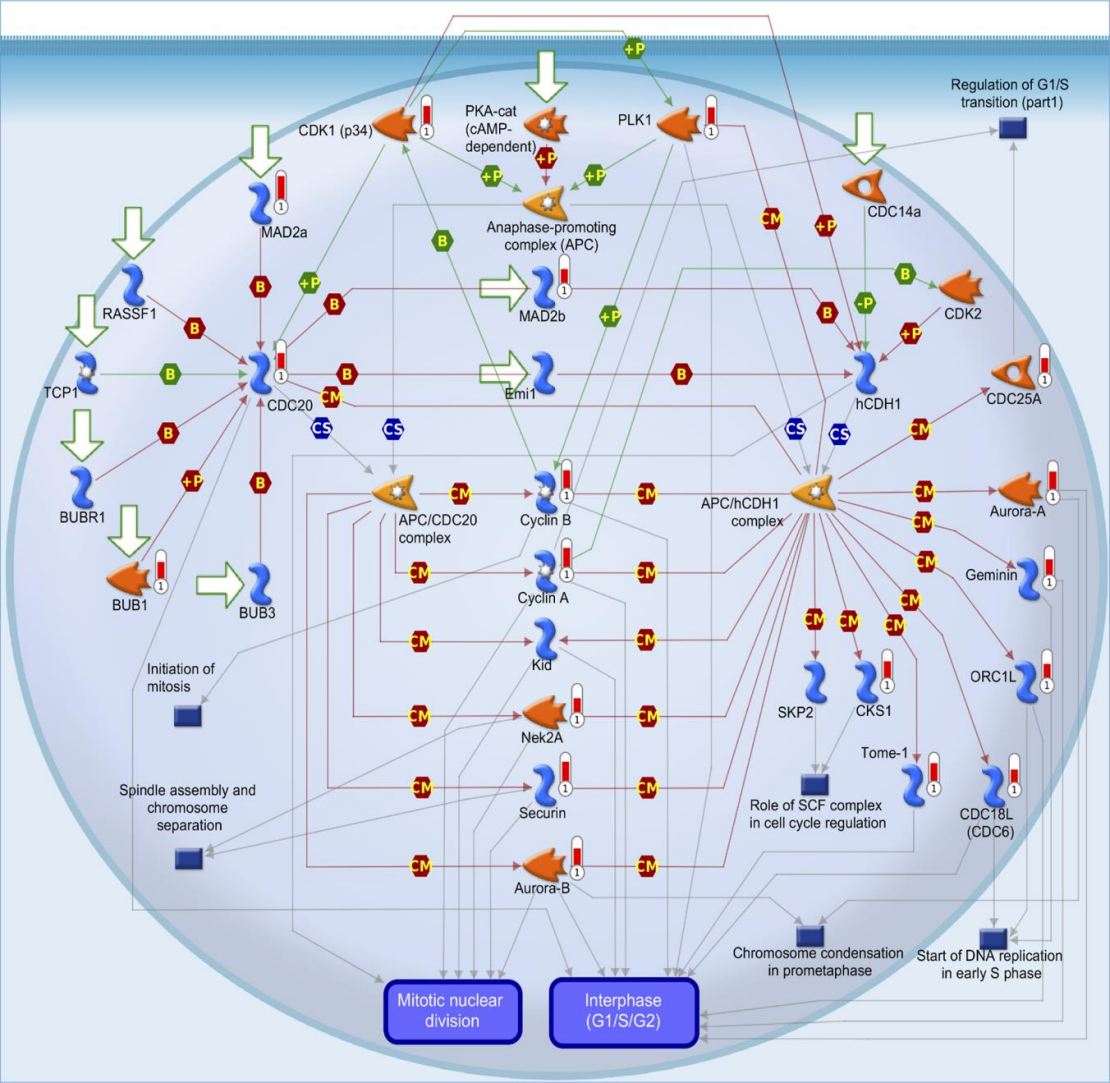
**MetaCore pathway analysis of coiled-coil domain-containing protein 167 (CCDC167)-co-expressed genes in breast cancer patient databases.** CCDC167-co-expressed genes in breast cancer from TCGA and METABRIC databases were acquired and identified by the Venn diagram analysis in Figure 2. These 945 genes were further exported to the MetaCore pathway analysis tool to identify gene networks and signaling pathways. The “cell cycle role of anaphase-promoting complex (APC) in cell cycle regulation” was the most significantly associated pathway. APC forms a complex with cell division cycle 20 (CDC20) or cadherin-1 (CDH1) to regulate the cell cycle.

**Figure 4:**
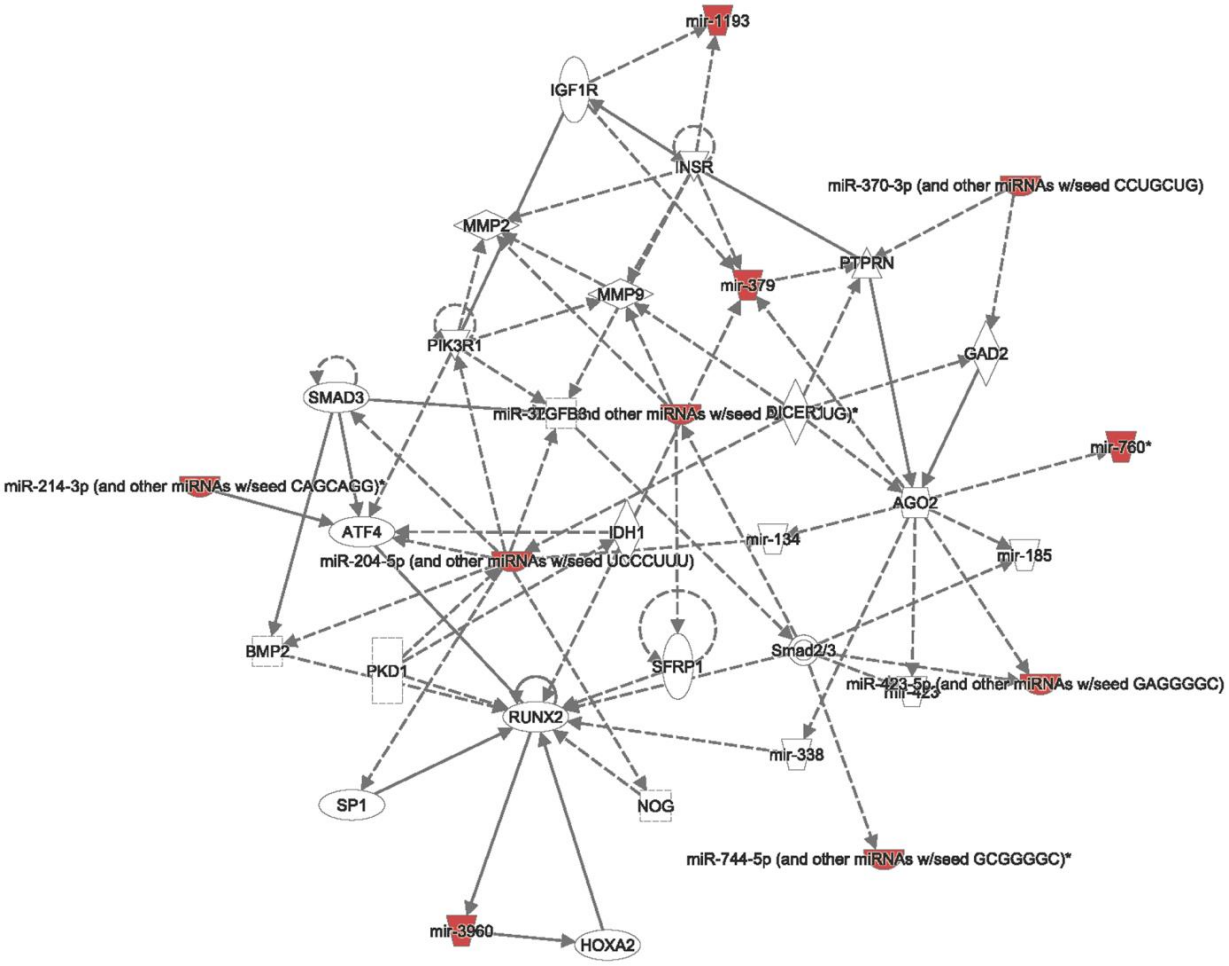
**Interacting networks between coiled-coil domain-containing protein 167 (CCDC167) and micro (mi)RNA.** The miRWalk database analyzed CCDC167-interacting miRNAs, and then the related networks were analyzed by an Ingenuity Pathway Analysis (IPA). These miRNAs and CCDC167-co-expressed genes are critical to the progression of breast cancer.

In addition, from both TCGA and METABRIC databases, we found that “cell cycle role of the anaphase-promoting complex (APC) in cell cycle regulation” was the most significantly regulated pathway by CCDC167 co-expression in breast cancer (Figure 3). We also identified several miRNAs through the miRWalk and IPA databases, including hsa-mir-760, hsa-mir-1193, hsa-mir-3960, hsa-miR-214-3p, hsa-miR-204-5p, hsa-miR-370-3p, hsa-miR-423-5p, and hsa-miR-744-5p, which may interact with CCDC167 (Figure 4).

### Downregulation of CCDC167 inhibits the growth of MCF-7 cells

**Figure 5:**
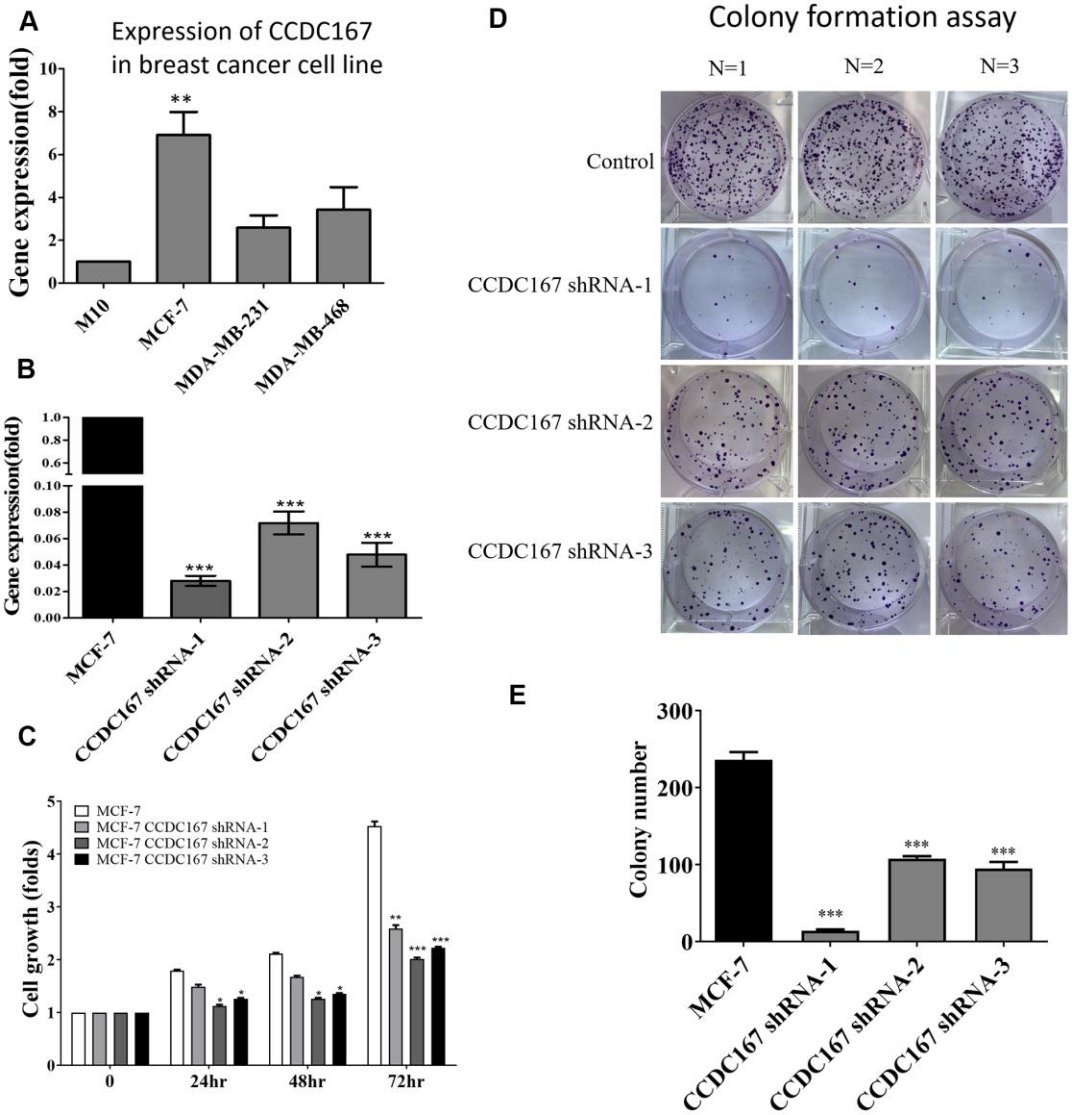
**Coiled-coil domain-containing protein 167 (CCDC167)-knockdown by shRNA significantly attenuated the proliferation of MCF-7 breast cancer cells.** (**A**) The mRNA expression of CCDC167 was determined in a variety of breast cancer cell lines. (**B**) A qPCR analyzed CCDC167 mRNA expression in shCCDC167-knockdown cells. (**C**) Evaluation of the growth of shCCDC167-knockdown and vector control MCF-7 cells according to MTT assays. (**D**) A colony-formation assay determined proliferation rates of the MCF-7 vector control and stable shCCDC167-knockdown MCF-7 cells. (**E**) Statistical data of the colony-formation assay. Values are the average of assays performed in triplicate. The standard deviation is displayed using error bars (*n*=3). * *p*<0.05.

We further investigated the effect of increased expression of CCDC167 on carcinogenesis-related processes, such as proliferation, migration, and anchorage-independent growth. M10 (non-tumorigenic), MCF-7 (ER^+^ subtype), MDA-MB-231 (triple-negative subtype), and MDA-MB-468 (highly invasive) cell lines are widely used for breast cancer research, and they represent different subtypes [32, 33]. Therefore, we chose these four cell lines for further study. Endogenous levels of CCDC167 differed in a variety of breast cancer cell lines. We used a qPCR to detect CCDC167 mRNA levels, and results showed the highest expression of CCDC167 in MCF-7 cells compared to other breast cancer cell lines (Figure 5A). Plasmids of CCDC167-shRNA and the vector control from RNAi Core were transfected into the MCF-7 cell line. The knockdown efficiency of shRNA on exogenous CCDC167 was examined by a qPCR. A significant reduction in CCDC167 mRNA expression was observed upon transfection with the shCCDC167 plasmid (Figure 5B).

In order to investigate the anti-proliferative effect of CCDC167 shRNA on human breast MCF-7 cancer cells, we first measured its cell proliferation ability. The MTT assay detected differences in short-term cell proliferation between CCDC167-knockdown and control MCF-7 cells (Figure 5C). The long-term ability of cell proliferation was examined by a colony-formation assay. Long-term cell growth was suppressed in CCDC167-shRNA cells (Figure 5D), and the difference compared to control cells was significant (Figure 5E). Meanwhile, the ability to form colonies was higher in CCDC167-overexpressing MCF-7 cells, and the results confirmed that overexpression of CCDC167 promoted cell proliferation (Supplementary Figure 2A). Knockdown of CCDC167 significantly altered cell cycle-related and apoptosis-related genes (Supplementary Figure 2B). Percentages of both early and late apoptosis increased after CCDC167-knockdown (Supplementary Figure 3). Meanwhile, in order to investigate gene expressions of other CCDC family members in the METABRIC database, we also compared mRNA expression levels between different subtypes of breast cancer including claudin-low, basal, Her2, luminal A, and luminal B relative to normal breast tissues (Supplementary Figures 4–10). Several CCDC members were significantly overexpressed in breast cancer subtypes, which might imply the high impact of CCDC family genes on tumor development.

### The survival status and clinical application of CCDC167

**Figure 6:**
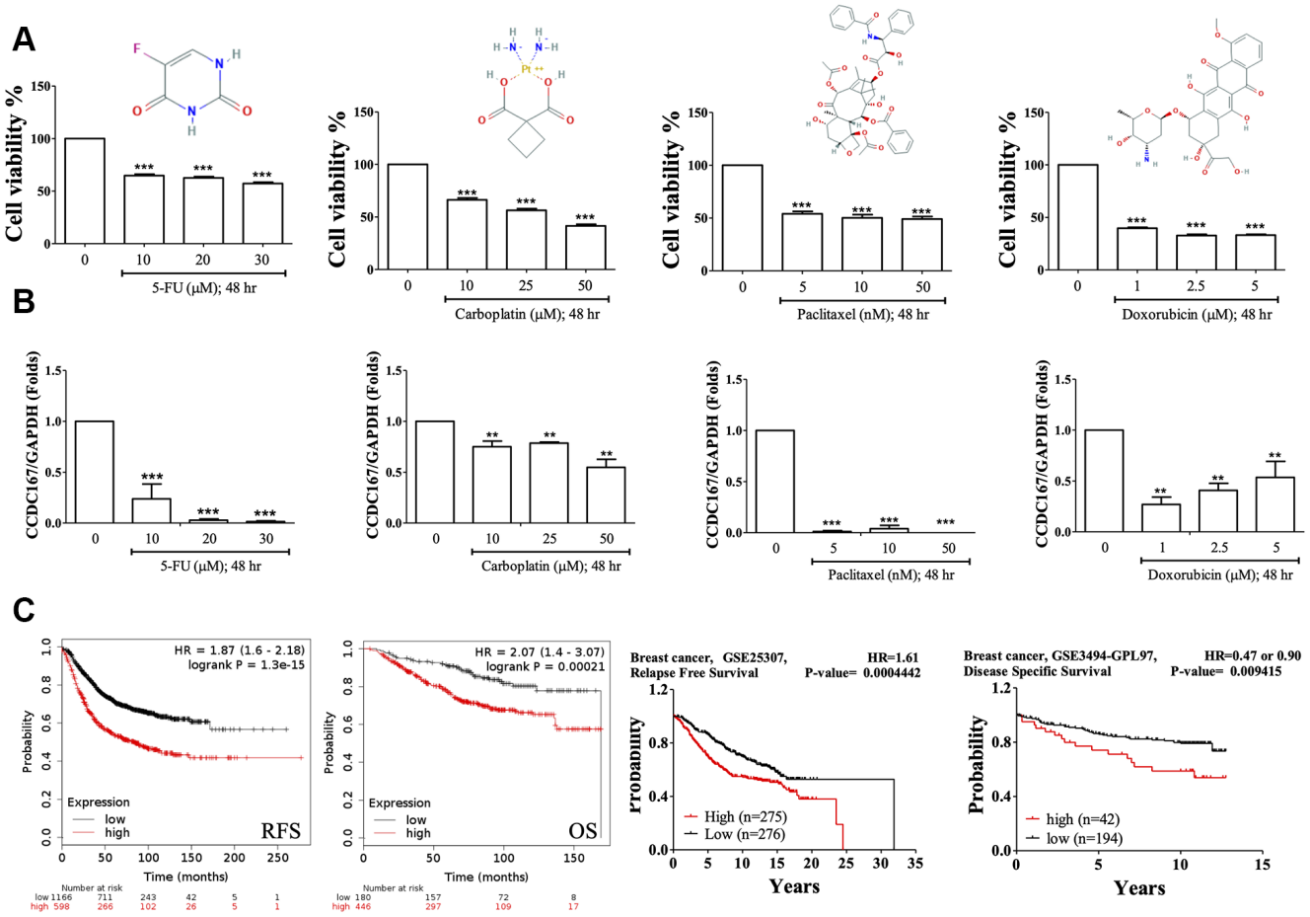
**Drug testing for breast cancer cell lines and the survival of breast cancer patients.** (**A**) Fluorouracil (5-Fu), carboplatin, paclitaxel, and doxorubicin treatments resulted in decreased cellular growths in MTT assays of MCF-7 cells. (**B**) Coiled-coil domain-containing protein 167 (CCDC167) mRNA alterations when treated with 5-Fu, carboplatin, paclitaxel, and doxorubicin for 2 days. (* *p*<0.05 was considered significant). (**C**) Correlation of CCDC167 recurrence-free survival and overall survival in Kaplan-Meier Plotter, using the GSE25307 dataset for recurrence-free survival and the GSE3494-GPL97 dataset for disease-specific survival in breast cancer patients. The red lines indicate high transcriptional expression levels of CCDC167, whereas the black lines indicate low expression levels. The plots also display hazard ratios (HRs), with 95% confidence intervals (CIs) and log-rank *p* values. High expression levels of CCDC167 predicted poor prognoses.

Fluorouracil (5-FU), carboplatin, paclitaxel, and doxorubicin are currently first- or second-line cytotoxic agents of adjuvant chemotherapy for breast cancer patients. Treatments with these compounds resulted in decreased cellular growth of MCF-7 cells (Figure 6A). Based on the qPCR results, we also found that treatment with these compounds also significantly decreased CCDC167 mRNA expression, which suggested that these drugs may downregulate CCDC167 signaling in breast cancer progression (Figure 6B).

We further analyzed CCDC167 overexpression in breast cancer patients (Figure 6C). Data on recurrence-free survival (RFS) and overall survival (OS) of breast cancer patients were collected from the Kaplan-Meier Plotter database. High expression of CCDC167 was correlated with worse RFS and OS. High expression of CCDC167 was also correlated with RFS in the GSE25307 datasets [34] and disease-specific survival in the GSE3494 datasets [35, 36]. All of these results demonstrated that high expression of CCDC167 predicted poor prognoses.

## DISCUSSION

Integrated analyses from this study showed that the CCDC167 expression level was higher in breast cancer. TCGA and METABRIC databases were used to analyze potentially CCDC167-co-expressed genes in breast cancer. We also used the MCF-7 breast cancer cell line and performed short-term and long-term cell proliferation assays to validate our bioinformatics predictions. CCDC167 expression has the potential to predict the survival of breast cancer patients and provide targets for further therapy. To the best of our knowledge, the present study is the first to provide comprehensive evidence of a novel association between CCDC167 and the prognosis of breast cancer patients.

The differential co-expression concept is related to gene-gene comparisons between two conditions, such as cancer and normal tissues. It implies that any alterations detected by conventional methods for differentially expressed genes might do not be beneficial if some genes not pass the differential threshold. However, insignificant changes in these genes could indirectly affect the regulatory system due to their co-expressed genes [37–39]. These differentially co-expressions could contribute to the disease without changing their expression levels. These differentially co-expressed genes and their associated disease can be revealed using gene-gene correlations between cancer and normal tissues. This information could potentially be useful in combination with conventional analyses of differential expression patterns. In addition, it could help identify underlying mechanisms in a biological system under different conditions. Detailed mechanisms of the CCDC167 gene in breast cancer were explored by examining its co-expressed genes from TCGA and METABRIC databases. Afterward, GeneGo and MetaCore annotations for enriched biological processes indicated that CCDC167-co-expressed genes were involved in cell cycle-related molecular processes. Next, in order to validate our bioinformatics predictions, we measured the ability of cells to proliferate with both a short-term MTT assay and a long-term colony-formation assay. MCF-7 shCCDC167 cells exhibited a significantly lower ability of cell proliferation compared to vector control cells.

Furthermore, we found that CCDC167-co-expressed genes were also involved in cell cycle and ubiquitination pathways, highlighting their essential roles in breast cancer. Cell division progression, ubiquinone metabolism, initiation of mitosis, spindle assembly, and chromosome separation were all top-ranked pathways of CCDC167-co-expressed genes. These results were found in the METABRIC and TCGA-BRCA datasets, and the findings were consistent across different populations. CCDC167-co-expressed genes were involved in the cell cycle, immune response, and ubiquitination-related pathways. CCDC167 also cooperated with several miRNA and oncogenic signaling pathways. The high expression of CCDC167 in breast cancer patients was also correlated with worse survival in these public databases. All of these data implicated the significance of CCDC167 in the progression of breast cancer. 5-FU, carboplatin, paclitaxel, and doxorubicin have the ability to inhibit breast cancer cell growth. These US FDA-approved compounds are currently first- or second-line regimens for postoperative adjuvant chemotherapy of breast cancer patients. We also found that treatment with fluorouracil, carboplatin, paclitaxel, and doxorubicin resulted in decreased expression of CCDC167 and suppressed growth of MCF-7 cells; these data suggested that CCDC167 signaling might be a critical target for these cytotoxic drugs. Other compounds that act against CCDC167 could be new therapeutic agents for treating breast cancer patients.

In conclusion, the present study demonstrated the crucial roles played by CCDC167 and its downstream signaling in breast cancer patients. These CCDC167-related pathways could potentially be targeted for treating and preventing breast cancer. The current study focused on CCDC167 pathways and cell cycle-related signatures, and these findings suggest that CCDC167 could have high potency as targeted therapy for breast cancer.

## MATERIALS AND METHODS

### Cell culture, and 3-(4,5-dimethylthiazol-2-yl)-2,5-diphenyltetrazolium bromide (MTT) and colony-formation assays

MCF-7 cells were grown in 10% fetal bovine serum (FBS) supplemented with Dulbecco’s modified Eagle’s medium (DMEM). The ability for short-term cell proliferation was examined by an MTT assay. A colony-formation assay was utilized to evaluate the ability of long-term cell proliferation. MCF-7 cells were trypsinized and seeded in six-well plates at a low density of 500 cells/well and grown in the presence of US Food and Drug Administration (FDA)-approved drugs predicted from a bioinformatics analysis [40–45]. After 2 weeks, the medium was aspirated from the six-well plates, and MCF-7 cells were fixed with methanol. Then, 0.1% crystal violet in distilled water was added to each well for 10 min to stain the colonies. After the crystal violet solution was removed, the plates were washed with distilled water five times and air-dried. Colonies in each well were enumerated under low-magnification light microscopy.

### Determination of mRNA expression using a reverse-transcription quantitative polymerase chain reaction (RT-qPCR)

We used TRIzol reagent (Invitrogen, Carlsbad, CA, USA) for total RNA extraction from MCF-7 cells following a datasheet. Next, a cDNA Reverse Transcription Kit (Applied Biosystems, Carlsbad, CA, USA) was used for reverse transcription of total RNA (1 μg) to complementary (c)DNA. A real-time PCR with the FastStart Universal SYBR Green Master (Roche, Basel, Switzerland) kit was conducted with 5% of each cDNA as the template with the ABI Step One Plus system (Applied Biosystems). Each experiment was repeated three times, and all results were normalized to GAPDH [46–51]. For small hairpin (sh)RNA-mediated signaling, shRNAs targeting CCDC167 and vector control were purchased from the National RNAi Core Facility (Academia Sinica, Taipei, Taiwan; http://rnai.genmed.sinica.edu.tw) according to accession no. TCRN0000140864 with target sequence 5’ -CCG GCC TAG TGT TCA AGC ATG GCT TCT CGA GAA GCC ATG CTT GAA CAC TAG GTT TTT TG-3’; no. TCRN0000142200 with target sequence 5’ -CCG GGA AGT TTC TTC GGC AAG AGA ACT CGA GTT CTC TTG CCG AAG AAA CTT CTT TTT TG-3’; and TCRN0000122824 with target sequence 5’ -CCG GGC CTA ATG AAC AAA GCC TCC ACT CGA GTG GAG GCT TTG TTC ATT AGG CTT TTT TG-3’. A control construct (pLKO.1 containing luciferase non-silenced shRNA) was also purchased from the National RNAi Core Facility as an expression control. The following human primers for the qPCR were used in this study: human CCDC167 forward 5’ -AGA CCT GGA GGC CGT GAA CT-3’ and reverse 5’ -AGA CCT GGA GGC CGT GAA CT-3’; GAPDH forward 5’-GAT TCC ACC CAT GGC AAA TTC-3’ and reverse 5’-AGC ATC GCC CCA CTT GAT T-3’; RIPK1 forward 5’-GCA CCG CTA AGA AGA ATG G -3’ and reverse 5’-GCC ACA CAA TCA AGT TGA AGA G-3’; Fas forward 5’-GAC CCA GAA TAC CAA GTG CAG-3’ and reverse 5’-GTT CTG CTG TGT CTT GGA CAT TGT C-3’; caspase-3 forward 5’-TGG CAT ACT CCA CAG CAC CTG GTT A-3’ and reverse 5’-CAT GGC ACA CAA AGC GAC TGG ATG AA-3’; cytochrome-c forward 5’-TTT GGA TCC AAT GGG TGA TGT TGA G-3’ and reverse 5’-TTT GAA TTC CTC ATT AGT AGC TTT TTT GAG-3’; Bax forward 5’-TGC TTC AGG GTT TCA TCC AG-3’ and reverse 5’-GGC GGC AAT CAT CCT CTG-3’; and tumor necrosis factor (TNF)-α forward 5’-TGC TTC AGG GTT TCA TCC AG-3’ and reverse 5’-GGC GGC AAT CAT CCT CTG-3’.

### Cell cycle assessment of CCDC167-knockdown in MCF-7 cellsq by flow cytometry

For the cell cycle assay, CCDC167 knockdown MCF-7 cells and the parental cells were grown in 10 cm dish, then we used trypsin to isolate cell. Next, the cells were centrifuged for 5 min at 1000×g, fixed with 70 % alcohol as well as ice-cold PBS, and incubated at 40° C for a minimum of 30 min. RNase (Takara, Shiga, Japan) was added to the samples within 30 min at room temperature. The propidium iodide (PI) solution and Annexin V-FITC Apoptosis Kit (AAT Bioquest, Inc. CA, USA) and was used for cell cycle experiments. All samples were analyzed using flow cytometry (FACS Calibur; BD Biosciences, San Jose, CA, USA) with a counting threshold of 10^6^ cells.

### Analyses of CCDC167 gene expression in multiple types of cancers in the Oncomine and gene expression profiling interactive analysis (GEPIA)

Transcriptomics expression levels of CCDC167 in multiple types and subtypes of cancers were screened in the Oncomine database with public high-throughput datasets using differential analysis options for cancer versus normal samples in the primary filters [52]. The method was clearly described in our previous studies [53–57]. Briefly, the thresholds for *p* values, multiples of change, and gene rank percentiles were set to 0.001, 1.5, and the top 10%, respectively. For the co-expression analysis, we selected breast cancer-related datasets which satisfied the thresholds mentioned above. The dataset with the highest correlation score was selected for analysis of genes co-expressed with CCDC167. In addition to the Oncomine database, we also used the GEPIA to analyze mRNA expression levels of CCDC167 in 33 datasets available in The Cancer Genome Atlas (TCGA) database [58]. CCDC167 expression levels were compared between tumor and normal groups, and these comparisons are illustrated in a dot plot with a log_2_ scale of transcripts per million (TPM) [59].

### Bioinformatics, functional enrichment analysis, and micro (mi)RNA-regulated networks

The RNA-Seq dataset from TCGA [60] and Molecular Taxonomy of Breast Cancer International Consortium (METABRIC) [61] were retrieved from the cBioPortal [62]. Normalized expression data were used to obtain the top 10% co-expressed genes based on CCDC167 expression, and a Venn diagram was plotted to determine genes highly correlated with CCDC167. Finally, these co-expressed genes were further analyzed using networks and pathways. The molecular functions and disease pathways of Gene Ontology (GO) terms in MetaCore (GeneGo, St. Joseph, MN, USA) were used to screen and analyze signaling networks in these breast cancer patients [63–66]. A *p* value of <0.05 represented statistical significance. To search for CCDC167-associated miRNAs, we used miRWalk 2.0 to predict potentially regulated miRNAs. Based on the miRmap score, which was calculated as the repressive strength of miRNA binding to its target mRNA, we chose a cutoff of the miRmap score of >99 for inclusion in our study and analyzed regulated pathways and networks with an Ingenuity Pathway Analysis (IPA).

### mRNA expressions of CCDC family members in different subtypes of breast cancer

Expression data from CCDC family genes were collected from the METABRIC database and plotted with a violin plot for comparisons among different molecular subtypes, including basal, claudin-low, HER-2, luminal A, luminal B, and normal-like breast cancers. Plots were conducted with R studio vers. 1.2.1335 under R vers. 4.0.3 using the ggpubr package vers. 0.4.0 [67].

### Analysis of survival probability

Correlations of mRNA expression levels of CCDC167 and breast cancer patient survival were analyzed using the Kaplan Meier-plot database [68]. In brief, distant metastasis-free survival (DMFS) was selected for CCDC167 with all default settings of the Kaplan Meier-Plot database to obtain the survival curve. In addition, survival data of the GSE25307 and GSE3494-GPL97 datasets were downloaded from the NCBI GEO and PrognoScan databases, and the survival risk was calculated using the Kaplan-Meier method with the log-rank test.

### Statistical analysis

All results are reported as the mean ± standard deviation (SD) with three or more replicates. Student’s *t*-test was used to calculate differences between groups with *p*<0.05 accepted as significant.

## Supplementary Material

Supplementary Figures

Supplementary Table 1
