# Supplementary material for: CCDC167 as a potential therapeutic target and regulator of cell cycle-related networks in breast cancer
Source: Aging (Albany NY). 2021 Jan 10;13(3):4157–81. doi: 10.18632/aging.202382 (PMC7906182; doi:10.18632/aging.202382)
Supplement: Supplementary Figures [file aging-13-202382-s001.pdf]

SUPPLEMENTARY FIGURES

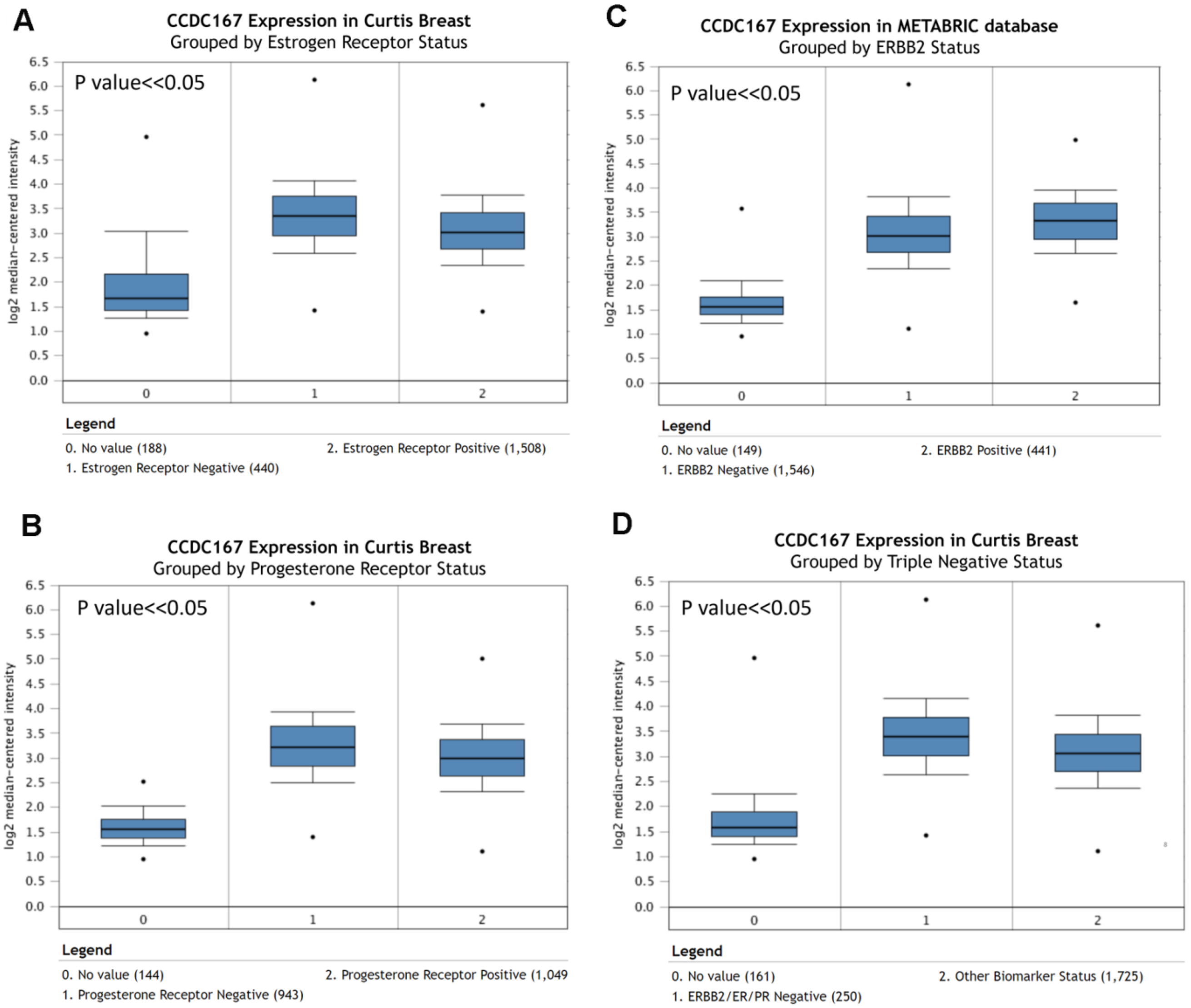

Supplementary Figure 1. Coiled-coil domain-containing protein 167 (CCDC167) expression in the METABRIC database (**A**) CCDC167 expression in estrogen receptor-positive (ER^+) breast cancer patients, (**B**) CCDC167 expression in progesterone receptor-positive (PR^+) breast cancer patients, (**C**) CCDC167 expression in $ERBB2^+$ (human epidermal growth factor receptor (HER)-2-enriched) breast cancer patients, and (**D**) CCDC167 expression in triple-negative breast cancer (TNBC) patients.

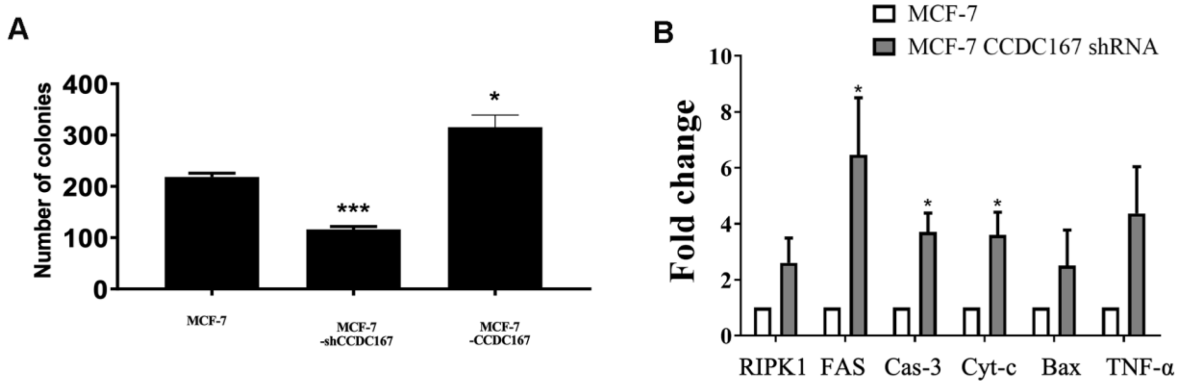

Supplementary Figure 2. Knockdown of coiled-coil domain-containing protein 167 (CCDC167) attenuates cellular growth by regulating cell cycle-related genes (**A**) Proliferation rates of the MCF-7 control cell line and shCCDC167-knockdown and CCDC167-overexpressing cells were determined by a colony-formation assay. (**B**) Knockdown of CCDC167 in MCF-7 cells increased apoptosis and necrosis-related gene expressions according to a qPCR.

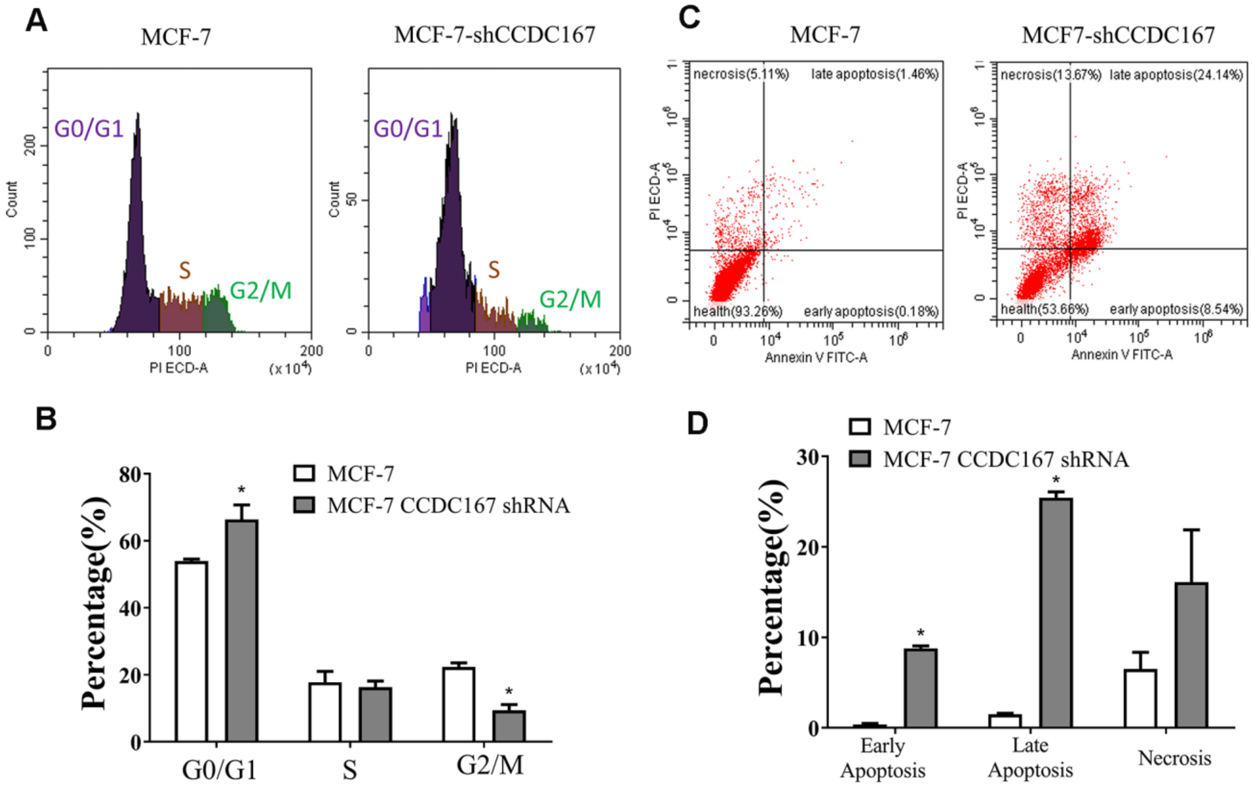

Supplementary Figure 3. Flow cytometric analysis of the cell cycle distribution and apoptosis. (**A**) The increment in the proportion of cells arrested in the G_0/G_1 phase after knockdown of coiled-coil domain-containing protein 167 (CCDC167) in MCF7 cells. (**B**) Statistical data of the cell cycle distribution from flow cytometry. (**C**) Knockdown of CCDC167 initiated early and late apoptotic states and necrotic states compared to the control group. (**D**) Statistical data of the apoptosis status from flow cytometry.

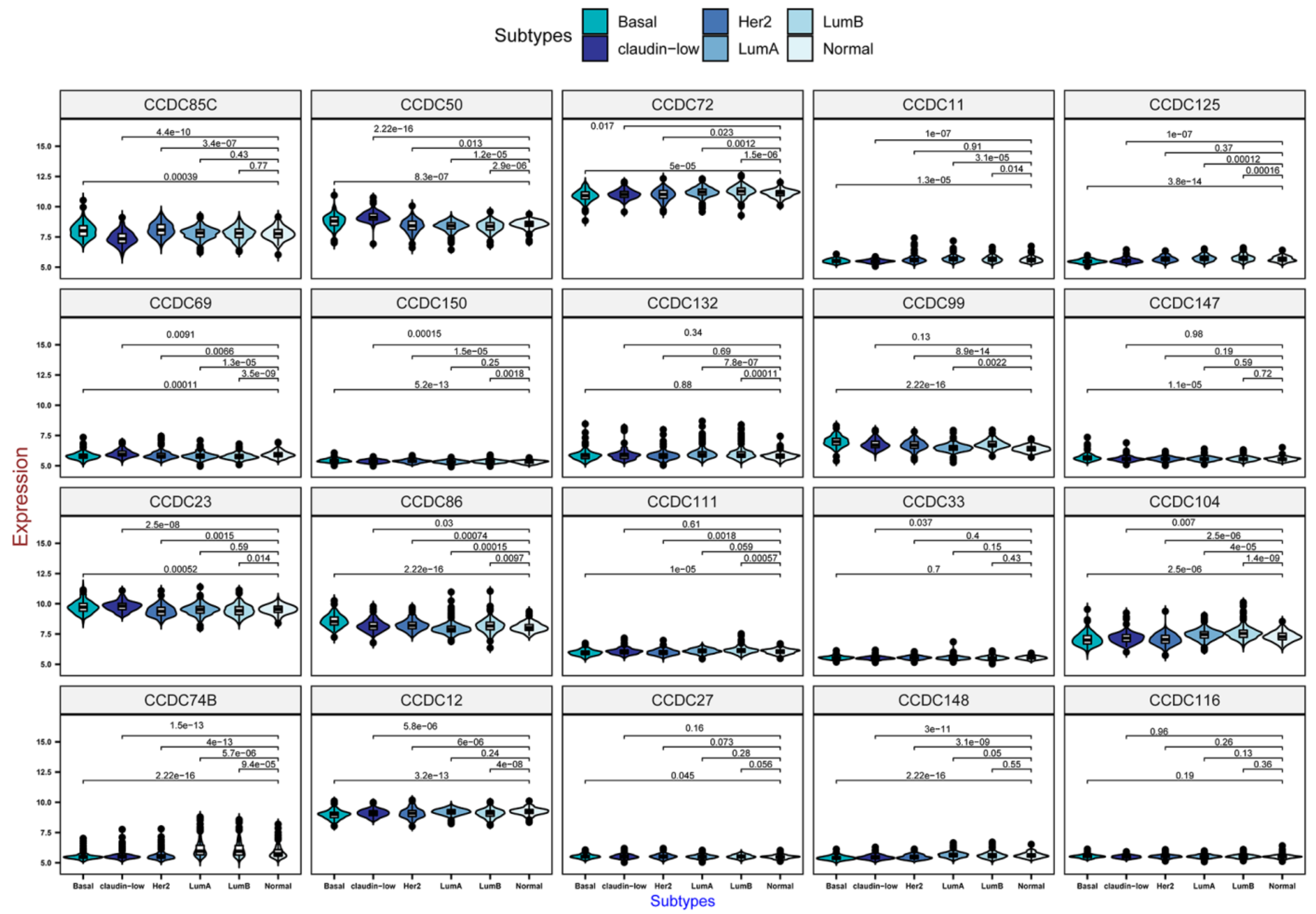

Supplementary Figure 4. Coiled-coil domain-containing protein (CCDC) family gene expressions in the METABRIC database. Comparison of members of the CCDC family in different subtypes of breast cancer (part-1).

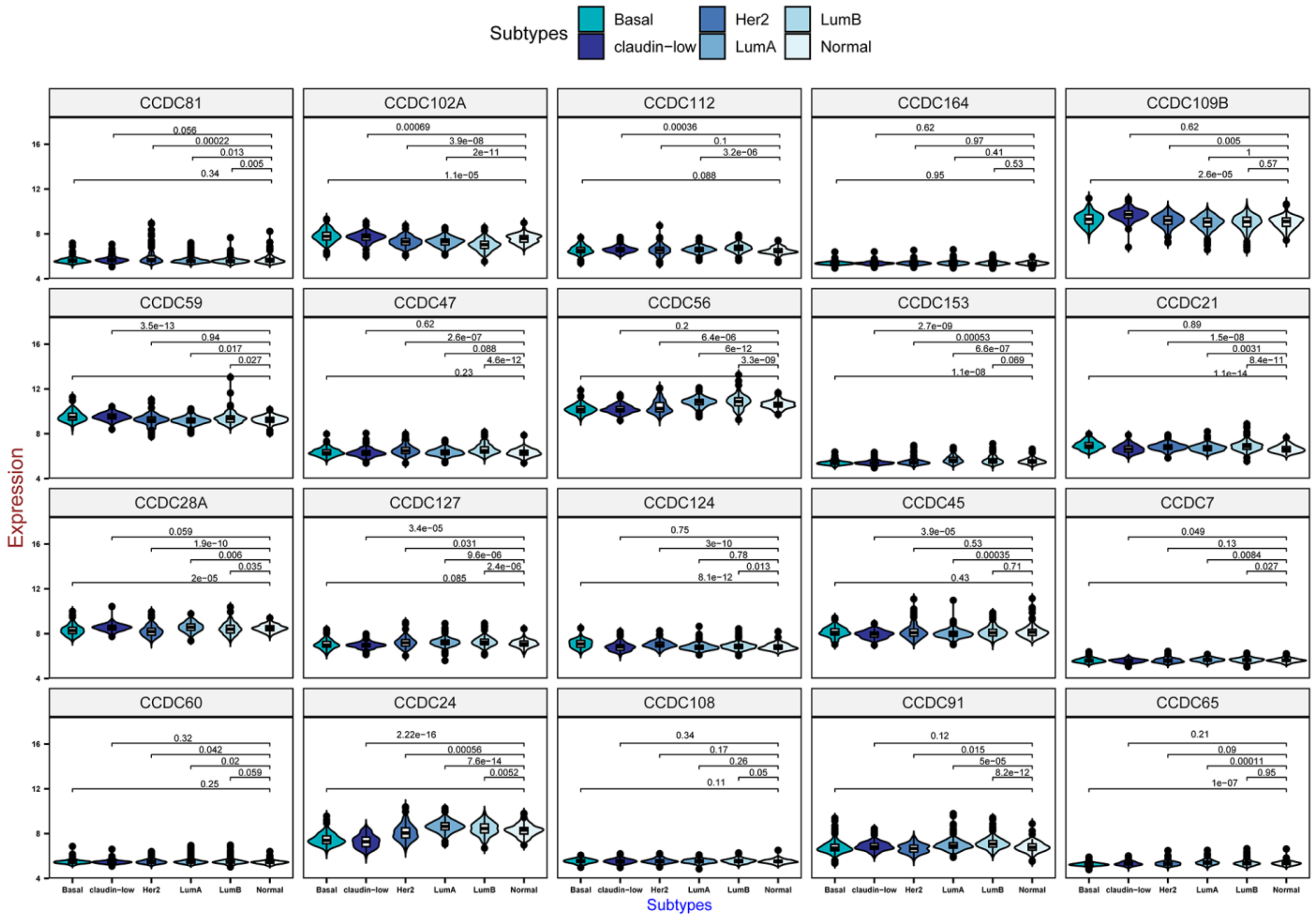

Supplementary Figure 5. The CCDC family gene expression in METABRIC database. Comparison of members of CCDC family in different subtypes of breast cancer (part-2).

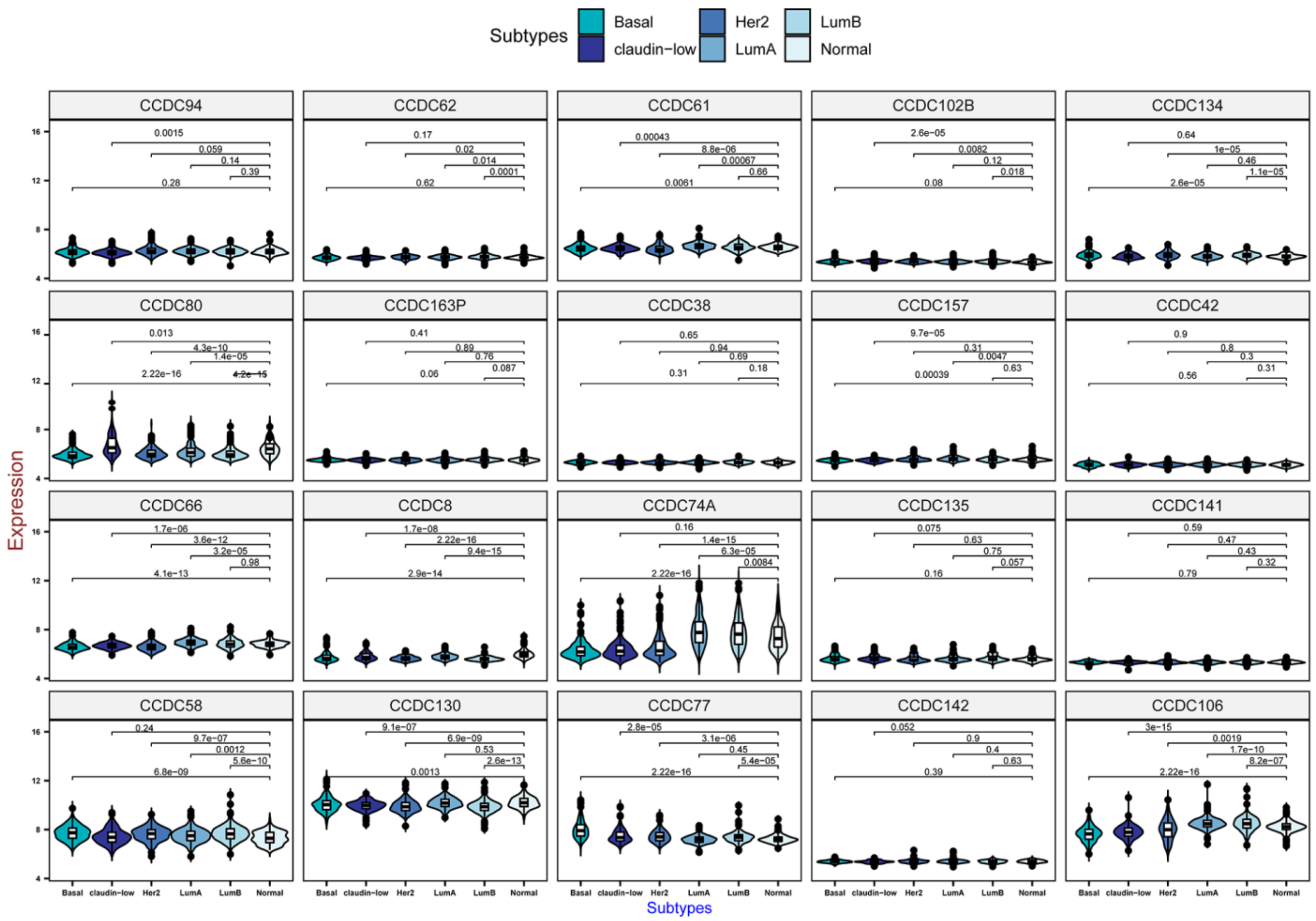

Supplementary Figure 6. The CCDC family gene expression in METABRIC database. Comparison of members of CCDC family in different subtypes of breast cancer (part-3).

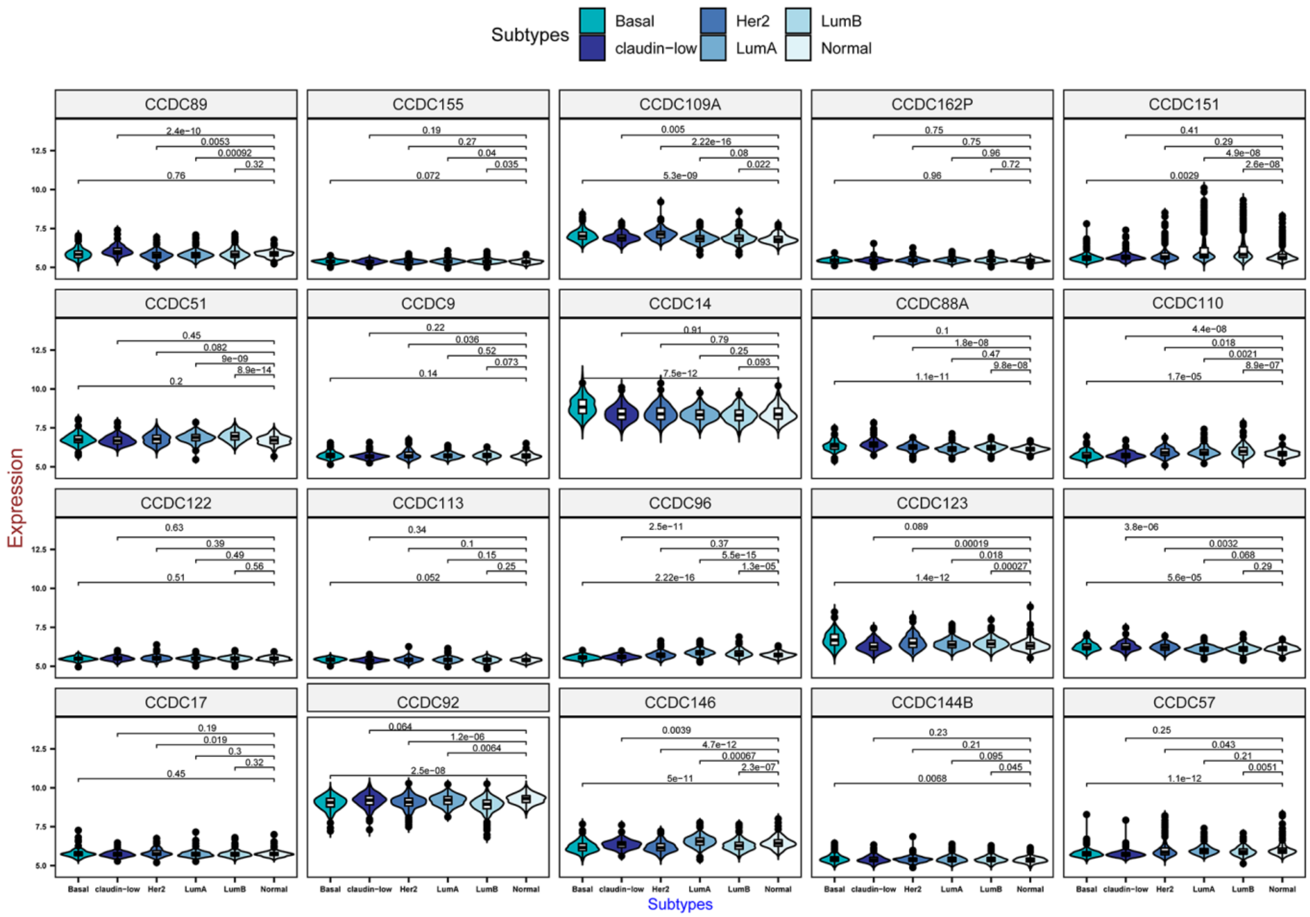

Supplementary Figure 7. The CCDC family gene expression in METABRIC database. Comparison of members of CCDC family in different subtypes of breast cancer (part-4).

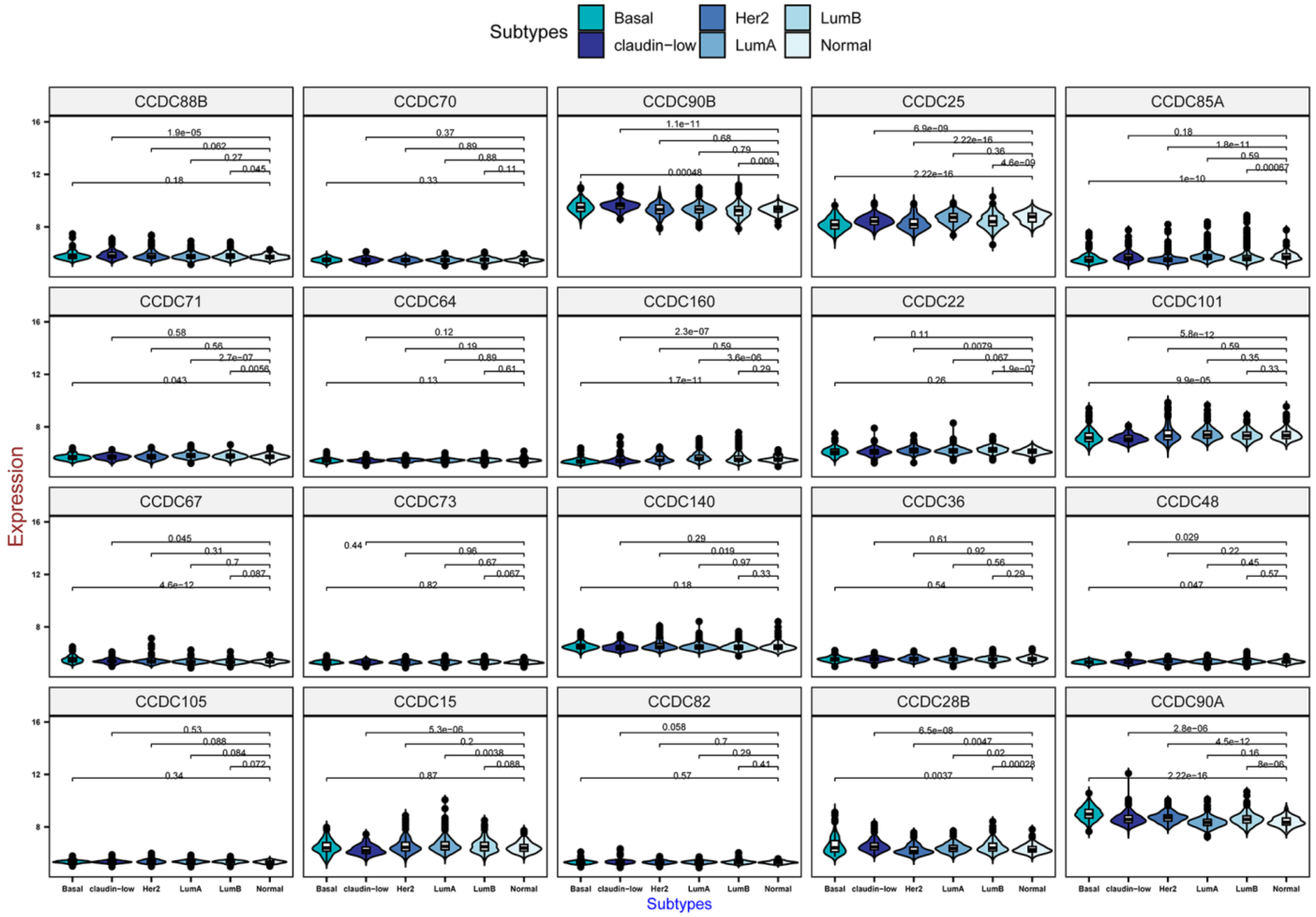

Supplementary Figure 8. The CCDC family gene expression in METABRIC database. Comparison of members of CCDC family in different subtypes of breast cancer (part-5).

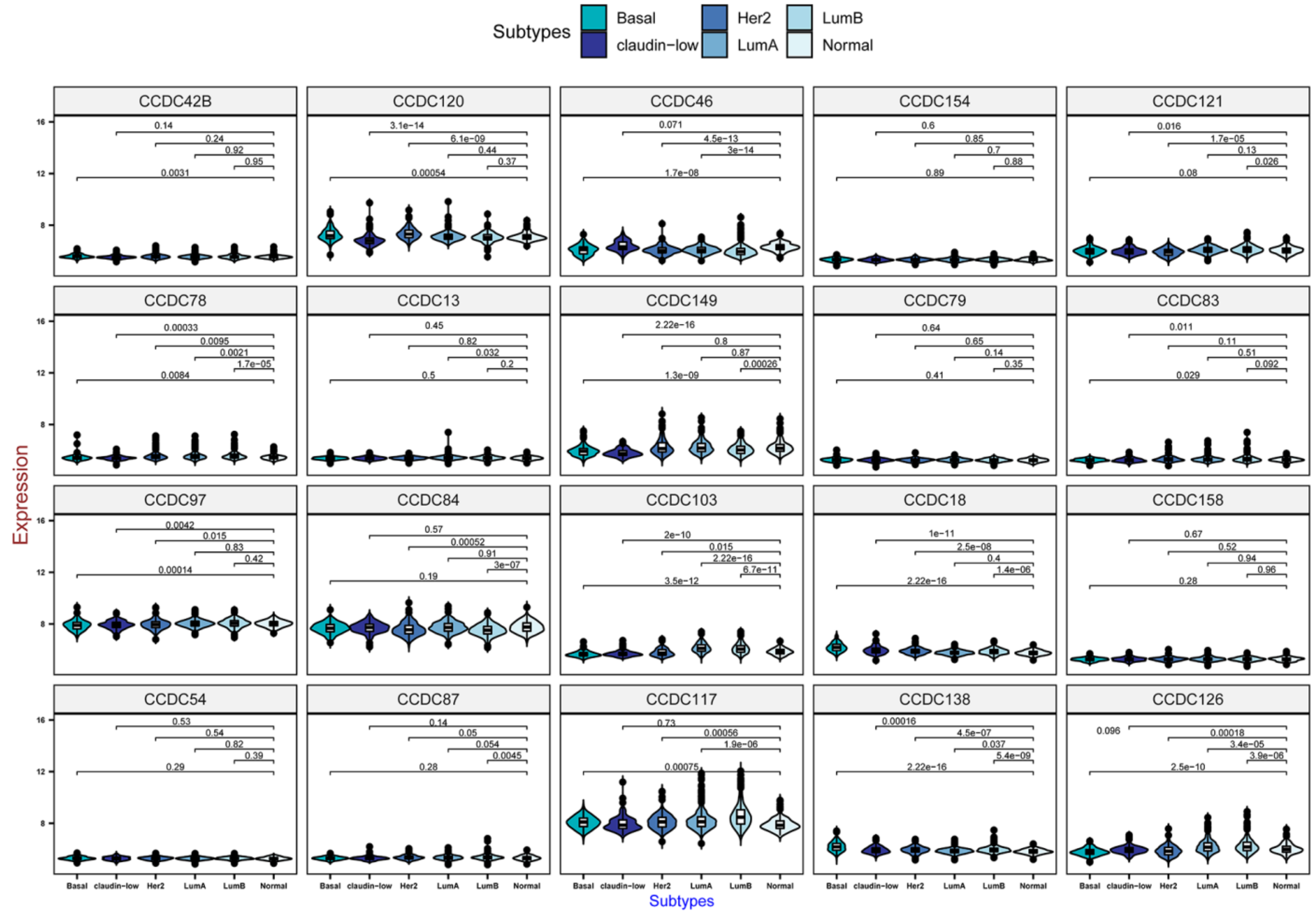

Supplementary Figure 9. The CCDC family gene expression in METABRIC database. Comparison of members of CCDC family in different subtypes of breast cancer (part-6).

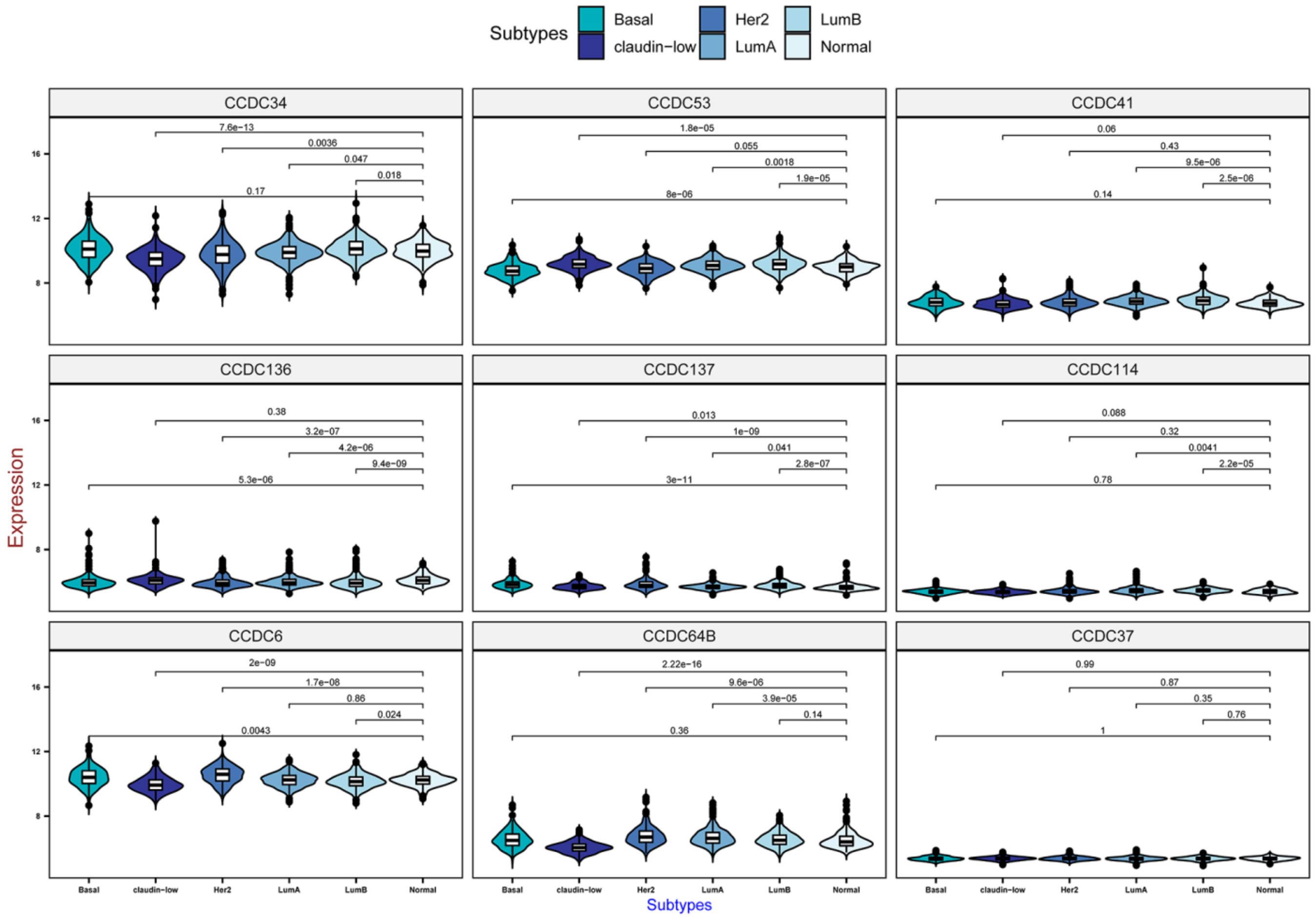

Supplementary Figure 10. The CCDC family gene expression in METABRIC database. Comparison of members of CCDC family in different subtypes of breast cancer (part-7).
