# Supplementary material for: CCDC167 as a potential therapeutic target and regulator of cell cycle-related networks in breast cancer
Source: Aging (Albany NY). 2021 Jan 10;13(3):4157–81. doi: 10.18632/aging.202382 (PMC7906182; doi:10.18632/aging.202382)
Supplement: Supplementary Table 1 [file aging-13-202382-s002.docx]

**Supplementary Table 1. Pathway analysis of 945 common coiled-coil domain-containing protein 167 (CCDC167)-co-expressed genes from TCGA and METABRIC databases using the MetaCore database (with *p*<0.01 set as the cutoff value).**

| Map | ***p* value** | **Genes in the present study** |
| --- | --- | --- |
| Cell cycle_Role of APC in cell cycle regulation | 1.12E-18 | MAD2b, CDC6, Tome-1, Cyclin A, Aurora-B, CDC25A, Cyclin B, ORC1L, CKS1, Nek2A, BUB1, Geminin, Aurora-A, PLK1, CDC20, MAD2a, Securin, CDK1 |
| Cell cycle_Spindle assembly and chromosome separation | 7.8E-17 | Importin (karyopherin)-alpha, RCC1, KNSL1, Aurora-B, HEC, Tubulin alpha, Cyclin B, Separase, Nek2A, TPX2, Aurora-A, CDC20, MAD2a, Securin, Ran, CDK1, Tubulin |
| Cell cycle_The metaphase checkpoint | 1.29E-14 | MAD2b, SPBC25, Aurora-B, HEC, HZwint-1, Survivin, CENP-E, Nek2A, BUB1, CENP-A, Aurora-A, PLK1, CDCA1, CDC20, CENP-F, MAD2a |
| Cell cycle_Role of Nek in cell cycle regulation | 7.88E-13 | Tubulin beta, Tubulin gamma, RCC1, HEC, Tubulin alpha, Histone H3, Nek2A, Cyclin B1, TPX2, Aurora-A, MAD2a, Ran, CDK1, Tubulin |
| Abnormalities in cell cycle in SCLC | 3.6E-12 | PCNA, Cyclin A, p14ARF, Aurora-B, E2F2, Histone H3, CKS1, CDK4, Cyclin B1, p16INK4, Cyclin E, CDK1, Cyclin E2 |
| Ubiquinone metabolism | 4.5E-11 | NDUFA8, NDUFA9, NDUFB7, NDUFB3, COQ3, NDUFB2, NDUFA11, coenzyme Q2 homolog, prenyltransferase, NDUFS3, DAP13, NDUFS5, NDUFAB1, NDUFA1, NDUFA4, NDUFB6, NDUFB5, NDUFC1, NDUFV3 |
| Cell cycle_Start of DNA replication in early S phase | 3.13E-10 | CDC18L, MCM3, ORC6L, CDC7, MCM2, ORC1L, MCM5, RPA3, Geminin, Cyclin E, MCM10, CDC45L |
| Cell cycle_Initiation of mitosis | 3.72E-10 | Lamin B, KNSL1, Cyclin B2, Histone H3, CDC25C, Cyclin B1, PLK1, CDC25B, FOXM1, Kinase MYT1, CDK1 |
| Cell cycle_Chromosome condensation in prometaphase | 5.69E-10 | CAP-H/H2, Cyclin A, Aurora-B, Cyclin B, TOP2, Histone H3, CAP-G, CAP-G/G2, Aurora-A, CDK1 |
| DNA damage_ATM/ATR regulation of G_2_/M checkpoint | 9.71E-10 | Chk2, Cyclin A, Chk1, Cyclin B, Cyclin B2, CDC25C, BLM, Cyclin B1, CDC25B, Kinase MYT1, CDK1 |
| Cell cycle_Cell cycle (generic schema) | 1.36E-08 | Cyclin A, CDC25A, Cyclin B, E2F2, CDC25C, CDK4, Cyclin E, CDC25B, CDK1 |
| ATP/ITP metabolism | 4.3E-08 | POLR2C, POLR2I, RRP41, RPB6, POLR2G, RPA16, RPA39, POLR2J, PM/SCL-75, NDPK A, RRM2, RRP46, HPRT, APRT, Small RR subunit, ADSL, RPB8, PNPH, ADA |
| GTP-XTP metabolism | 6.75E-08 | KGUA, POLR2C, POLR2I, RRP41, RPB6, POLR2G, RPA16, GMPS, RPA39, POLR2J, PM/SCL-75, NDPK A, RRP46, HPRT, RPB8, PNPH |
| Cell cycle_Role of SCF complex in cell cycle regulation | 3.61E-07 | Chk1, CDC25A, RING-box protein 1, CKS1, CDK4, Cyclin E, PLK1, NEDD8, CDK1 |
| Transcription_Negative regulation of HIF1A function | 5.78E-07 | MCM3, p14ARF, MCM7, ARD1, MCM2, PSMA7, MCM5, HSP90, PRDX4, RUVBL2, Sirtuin7, HSP90 beta, Elongin C |
| DNA damage_ATM/ATR regulation of G_1_/S checkpoint | 9.22E-07 | PCNA, Chk2, I-κB, Cyclin A, Chk1, CDC25A, CDK4, BLM, Cyclin E |
| Transport_RAN regulation pathway | 1.31E-06 | Importin (karyopherin)-alpha, RCC1, NUP62, NTF2, SUMO-1, RanBP1, Ran |
| Cell cycle_Transition and termination of DNA replication | 1.77E-06 | TOP2 alpha, PCNA, Cyclin A, MCM2, TOP2, FEN1, POLD cat (p125), CDK1 (p34) |
| Cell cycle_Nucleocytoplasmic transport of CDK/Cyclins | 3.99E-06 | Importin (karyopherin)-alpha, Cyclin A, CDK4, Cyclin B1, Cyclin E, CDK1 |
| Cell cycle_Sister chromatid cohesion | 6.25E-06 | PCNA, Cyclin B, Separase, Histone H3, DCC1, Securin, CDK1 (p34) |
| Cell cycle_Role of 14-3-3 proteins in cell cycle regulation | 6.25E-06 | Chk2, Chk1, CDC25A, CDC25C, 14-3-3 zeta/delta, CDC25B, CDK1 |
| Reproduction_Progesterone-mediated oocyte maturation | 7.02E-06 | CDC25C, BUB1, Cyclin B1, Aurora-A, PLK1, CDC20, CDC25B, Kinase MYT1, CDK1 |
| Cytoskeleton remodeling_Neurofilaments | 1.61E-05 | Tubulin beta, Tubulin gamma, Actin cytoskeletal, CDK5, Tubulin alpha, Tubulin gamma 1, Tubulin |
| CTP/UTP metabolism | 1.89E-05 | CTP synthase, POLR2C, POLR2I, RRP41, RPB6, POLR2G, RPA16, RPA39, POLR2J, PM/SCL-75, NDPK A, UCK2, RRP46, RPB8 |
| Immune response_IFN-alpha/beta signaling via PI3K and NF-κB pathways | 2.05E-05 | PCNA, 4E-BP1, I-κB, Cyclin A, p70 S6 kinases, CDC25A, ISG15, CDK4, p16INK4, Cyclin E, p19, eIF4A, CDK1 |
| dATP/dITP metabolism | 2.05E-05 | KGUA, POLA2, 8ODP, POLE4, DNA polymerase theta, POLE2, POLD cat, NDPK A, RRM2, Small RR subunit, ADSL, PNPH, ADA |
| Cell cycle_Regulation of G_1_/S transition (part 1) | 3.84E-05 | Chk2, PP2A regulatory, Cyclin A, CDC25A, RING-box protein 1, CDK4, p16INK4, Cyclin E |
| Stem cells_H3K36 demethylation in stem cell maintenance | 4.31E-05 | EZH2, p14ARF, M33, Histone H3, CDK4, p16INK4 |
| Regulation of degradation of deltaF508-CFTR in CF | 4.69E-05 | RNF5, UFD1, HSP90, Sti1, Aha1, SAE1, Derlin1, HSPBP1 |
| Apoptosis and survival_Role of IAP-proteins in apoptosis | 7.35E-05 | HtrA2, Bax, Survivin, Aif, Cyclin B1, Smac/Diablo, CDK1 |
| Apoptosis and survival_Endoplasmic reticular stress response pathway | 0.000119 | I-κB, Bax, C/EBP zeta, Bak, PP1-cat, Endoplasmin, Derlin1, PP1-cat alpha, ERP5 |
| Cell cycle_ESR1 regulation of G_1_/S transition | 0.000167 | Cyclin A, CDC25A, CKS1, CDK4, Cyclin A2, NCOA3, Cyclin E |
| Putative pathways of activation of monoclonal protein secretion in multiple myeloma | 0.00017 | SSR-delta, DNAJB11, Cyclophilin B, DAD-1, ARMET, ERP5 |
| dCTP/dUTP metabolism | 0.000224 | POLA2, POLE4, DNA polymerase theta, POLE2, POLD cat, NDPK A, RRM2, TK1, Small RR subunit, NT5C3 |
| dGTP metabolism | 0.000339 | KGUA, POLA2, 8ODP, POLE4, DNA polymerase theta, POLE2, POLD cat, NDPK A, PNPH |
| Transcription_Role of heterochromatin protein 1 (HP1) family in transcriptional silencing | 0.000398 | CDC25A, Histone H3, HDAC2, SUMO-1, Cyclin A2, Cyclin E, CDK1 |
| Histone deacetylases in prostate cancer | 0.000405 | Bax, Tubulin alpha, HDAC2, HSP90, Sirtuin5, Sirtuin7 |
| Transcription_Ligand-dependent activation of the ESR1/SP pathway | 0.000492 | CDC25A, NCOA3 (pCIP/SRC3), TYSY, Cyclin E, Cyclin E2, ADA |
| Development_Slit-Robo signaling | 0.000492 | Profilin I, Actin cytoskeletal, CDK5, Actin, ACTB, Tubulin |
| Regulation of degradation of wtCFTR | 0.000494 | RNF5, UFD1, HSP90, Derlin1, HSPBP1 |
| Role of XBP1 protein in multiple myeloma | 0.000494 | DNAJB11, PSMA7, PSMA5, PSMA6, ERP5 |
| Epigenetic alterations in ovarian cancer | 0.000521 | DNMT3B, EZH2, Bax, Aurora-B, Histone H3, HDAC2, CDK4, p16INK4, Aurora-A, CDC20 |
| TTP metabolism | 0.000536 | POLA2, Thymidylate kinase, POLE4, DNA polymerase theta, POLE2, POLD cat, NDPK A, TYSY, TK1 |
| Oxidative stress_Role of ASK1 under oxidative stress | 0.000572 | Thioredoxin, SOD1, HPK38, PRDX1, MT-TRX, 14-3-3 zeta/delta, Glutaredoxin, 14-3-3 |
| Inhibition of tumor suppressive pathways in pancreatic cancer | 0.00063 | Bax, p14ARF, CDK4, p16INK4, Rad51 |
| Possible regulation of HSF-1/chaperone pathway in Huntington's disease | 0.00063 | HSF1, HSP90, PLA2, PLK1, HSP90 beta |
| Inhibition of remyelination in multiple sclerosis: regulation of cytoskeleton proteins | 0.000726 | Tubulin beta, Actin cytoskeletal, CDK5, Tubulin alpha, Stathmin, Tubulin beta 4, Tubulin |
| Apoptosis and survival_Regulation of apoptosis by mitochondrial proteins | 0.00084 | Bax, VDAC 2, Aif, Bak, Smac/Diablo, ANT |
| Cytoskeleton remodeling_Regulation of actin cytoskeleton nucleation and polymerization by Rho GTPases | 0.000955 | DRF, Actin cytoskeletal, Profilin, mDIA2, Rac1-related, Rac3, F-Actin cytoskeleton |
| Cytoskeleton remodeling_Keratin filaments | 0.001354 | Tubulin beta, Actin cytoskeletal, Tubulin alpha, Tubulin gamma 1, CDK1, Tubulin |
| Immune response_IL-4-induced regulators of cell growth, survival, differentiation, and metabolism | 0.001428 | MCM6, Cyclin A, Bax, CDC25A, MCM5, CDK4, Cyclin E, Cathepsin V |
| Apoptosis and survival_DNA-damage-induced apoptosis | 0.001438 | Histone H2AX, Chk2, Chk1, BLM |
| IL-6 signaling in colorectal cancer | 0.001569 | Bax, Cyclin B, Survivin, Cyclin B1, Cyclin E, CDK1 |
| Nicotine/nAChR alpha-3/nAChR beta-2 signaling in NSCLC | 0.001766 | CDC18L, CDC25A, E2F2, Survivin, TYSY |
| Cell cycle progression in prostate cancer | 0.002077 | 4E-BP1, CDC25A, Cyclin B, CDK4, CDC25B, CDK1 |
| Immune response_Antigen presentation by MHC class I, classical pathway | 0.002492 | PSMB1, PSMB5, PSME2, Endoplasmin, Calreticulin, Impas 1, PSMB2 |
| Neurophysiological process_Dynein-dynactin motor complex in axonal transport in neurons | 0.002492 | Importin (karyopherin)-alpha, HAP40, CDK5, DYNLL, Tctex-1, DYNLT, Tubulin |
| Mitogenic action of estradiol/ESR1 (nuclear) in breast cancer | 0.002492 | CDC25A, CDK4, NCOA3, Cyclin E, Cyclin E2 |
| Apoptosis and survival_BAD phosphorylation | 0.003061 | Bax, PP2C, p70 S6 kinase2, PP1-cat alpha, CDK1, 14-3-3 |
| Role of histone modifications in progression of multiple myeloma | 0.003414 | EZH2, Tubulin alpha, Histone H3, HDAC2, CDK4 |
| Apoptosis and survival_Granzyme A signaling | 0.003414 | HMG2, NDUFS3, Histone H3, NDPK A, Lamin B1 |
| Signal transduction_AKT signaling | 0.003455 | PCNA, 4E-BP1, I-kB, Bax, RHEB2, HSP90 |
| Cigarette smoke-induced oxidative stress and apoptosis in airway epithelial cells | 0.004551 | Thioredoxin, Bax, C/EBP zeta, PRDX5, SOD1, PRDX1, Glutaredoxin |
| p53 signaling in prostate cancer | 0.005223 | EZH2, Bak, NK31, Stathmin, Tubulin |
| LRRK2 in neurons in Parkinson's disease | 0.005223 | Actin cytoskeletal, HSP90, ACTB, Tubulin, 14-3-3 |
| Role of Apo-2L(TNFSF10) in prostate cancer cell apoptosis | 0.005952 | I-kB, Bax, Survivin, Bak, Smac/Diablo |
| Development_Role of CDK5 in neuronal development | 0.005952 | Actin cytoskeletal, CDK5, Pin1, APOER2, Tubulin |
| HCV-mediated liver damage and predisposition to HCC via cell stress | 0.005967 | Bax, C/EBP zeta, HSP60, SOD1, Bak, Endoplasmin, Calreticulin |
| Microsatellite instability in gastric cancer | 0.006358 | PCNA, Bax, EXO1, p16INK4 |
| Higher ESR1/ESR2 ratio in breast cancer | 0.007621 | CDC25A, Cyclin A2, Cyclin B1, Cyclin E, FOXM1 |
| CFTR folding and maturation (normal and CF) | 0.008746 | Sti1, Aha1, HSP90 beta, HSPBP1 |
| Notch signaling in breast cancer | 0.009739 | Cyclin A, Bax, Pin1, HURP, Survivin, Cyclin B1 |
| TLRs-mediated IFN-alpha production by plasmacytoid dendritic cells in SLE | 0.009739 | La protein, 4E-BP1, SNRPB, SNRPD1, p70 S6 kinase2, IRAK1 |
